# One-year clinical follow-up of granulomatous lymphadenitis diagnosed via EBUS-TBNA in a tuberculosis-endemic region

**DOI:** 10.55730/1300-0144.6006

**Published:** 2025-04-07

**Authors:** Hasret Gizem KURT, Ayperi ÖZTÜRK, Melahat UZEL ŞENER, Figen ÖZTÜRK ERGÜR, Zeynep Tilbe SAYMAZ GUVANJOV, Aydın YILMAZ

**Affiliations:** 1 Department of Pulmonology, Private Sincan Koru Hospital, Ankara, Turkiye; 2 Division of Interventional Pulmonology, Department of Pulmonology, Faculty of Medicine, Health Sciences University Ankara Atatürk Sanatorium Training and Research Hospital, Ankara, Turkiye; 3 Department of Pulmonology, Sandwell and West Birmingham Nhs Trust, Birmingham, UK

**Keywords:** Endobronchial ultrasound-guided transbronchial needle aspiration, granulomatous lymphadenitis, sarcoidosis, sarcoid-like reaction, tuberculosis lymphadenitis

## Abstract

**Background/aim:**

Granulomatous lymphadenitis is not a specific clinical diagnosis. In regions where tuberculosis (TB) is endemic, differentiating between various diseases presenting with granulomatous lymphadenitis poses a significant clinical challenge. This study aims to evaluate the etiological distribution of underlying conditions and to assess diagnosis changes observed during at least one year of follow-up in patients diagnosed with granulomatous lymphadenitis through endobronchial ultrasound-guided transbronchial needle aspiration (EBUS-TBNA).

**Materials and methods:**

A total of 4711 patients were included in the study, and 9353 lymph node samples were collected. Granulomatous lymphadenitis was identified in 791 patients, from whom 1505 lymph node samples were obtained. A cohort of 453 patients was monitored for at least 1 year, during which 873 lymph node samples were collected. The medical records of these patients were retrospectively reviewed in detail, and the final clinical diagnosis for each patient was established at the conclusion of the 1-year follow-up period.

**Results:**

Sarcoidosis was the most common final diagnosis, accounting for 52.3% of cases, while tuberculosis lymphadenitis was diagnosed in 42.6% of patients. Diagnostic procedures, including acid-fast bacteria (AFB) staining, culture, and TB-PCR, were performed in 94.3% of the cohort. Nonnecrotizing granulomatous lymphadenitis was identified in 8 patients with a history of extrathoracic malignancy; 5 were diagnosed with sarcoid-like reactions and 3 with TB lymphadenitis. Additionally, during the 1-year clinical follow-up period, the initial diagnosis was revised in 14 patients.

**Conclusion:**

Long-term follow-up of clinical progression and treatment response is crucial for precise diagnosis and management. The study findings suggest that routine TB-PCR and AFB testing on EBUS-TBNA-derived lymph node samples could enhance diagnostic precision.

## 1. Introduction

Granuloma formation represents a chronic inflammatory response, characterized by the accumulation of modified macrophages surrounded by a neural network of epithelial cells and multinucleated giant cells [1]. Granulomatous lymphadenitis can be caused by various etiologies, such as sarcoidosis, sarcoid-like reactions (SLR), tuberculosis (TB), nontuberculous mycobacteria, fungi, parasites, viruses, vasculitis, and autoimmune diseases [2,3]. Endobronchial ultrasound-guided transbronchial needle aspiration (EBUS-TBNA) is a minimally invasive procedure with high diagnostic accuracy, commonly used for diagnosing and staging lung cancer, as well as detecting benign mediastinal or hilar pathologies, such as granulomatous lymphadenitis [4,5]. However, distinguishing between the potential etiologies of granulomatous lymphadenitis represents a considerable challenge, particularly in areas where TB is endemic, such as Türkiye, where the annual incidence of TB was 10.9 per 100.000 in 2023.^1^

In this study, the etiological distribution of patients with granulomatous lymphadenitis diagnosed via EBUS-TBNA was examined. The primary aim was to highlight the importance of clinical monitoring for at least 1 year after diagnosis, as changes in diagnosis may occur due to lack of clinical or radiological response to treatment, or relapse.

## 2. Materials and methods

This study was conducted retrospectively with the approval of the Ankara Atatürk Sanatorium Training and Research Hospital Ethics Committee, granted on 12 April 2022 (Approval No: 2012 – KAEK – 15 / 2496). Patients included in the study were those diagnosed histopathologically with granulomatous lymphadenitis via EBUS-TBNA between 1 January 2013 and 31 December 2020, and who had been clinically followed for at least 1 year at our institution. The number of samples and location of lymph nodes were documented. Lymph nodes with granulomatous lymphadenitis were categorized as either necrotic or nonnecrotic. The results of acid fast bacteria (AFB) staining, TB-polymerase chain reaction (PCR), and TB cultures from the biopsy material were also recorded. Additionally, the clinical history, medical records within 1 year after EBUS-TBNA, treatment response, and follow-up outcomes of patients diagnosed with granulomatous lymphadenitis were thoroughly reviewed, and the final clinical diagnoses were documented.

### 2.1. EBUS procedure

The EBUS-TBNA test was performed in an operating room by experienced interventional pulmonologists using a convex EBUS probe (BF-UC180F; Olympus, Tokyo, Japan) with a dedicated scanner (EU-ME1; Olympus, Tokyo, Japan) in patients under deep sedation with midazolam and propofol. At least two lymph nodes were sampled for an average of three times. No major complications were reported.

### 2.2. Cytological examination

Cell blocks and cytological samples were prepared for each sampled lymph node. The cytological material was first air-dried and then stained with May–Grünwald–Giemsa, hematoxylin and eosin before examination. Cell blocks were prepared by rinsing the material with 10 mL of saline. They were then immediately sent to the pathology department. However, a portion of each sample was reserved for the identification of *Mycobacterium tuberculosis* using direct microscopy, Lowenstein–Jensen culture, and the GeneXpert test (replacing the previously used PCR method).

### 2.3. Histopathological classification of granulomatous inflammation

The definitive diagnosis of granulomatous inflammation requires specific histopathological features. These include large, elongated, irregular nuclei, finely granular chromatin, inconspicuous nucleoli, moderate amounts of pale to eosinophilic cytoplasm, and syncytial arrangements with indistinct cell borders. Furthermore, lymph nodes with granulomatous inflammation are histopathologically classified based on the presence or absence of necrosis.

### 2.4. Inclusion criteria for sarcoidosis

-Negative TB test results, including AFB staining, culture, and PCR tests
- Radiological and clinical findings compatible with sarcoidosis
- Clinical and radiological response following glucocorticoid therapy

### 2.5. Inclusion criteria for TB

- Positive AFB staining, PCR, or culture tests
- Histopathological diagnosis with granulomatous inflammation with caseating necrosis
- Clinical and radiological findings compatible with TB
- Clinical and radiological improvement following antituberculosis treatment

### 2.6. Inclusion criteria for SLR

- Absence of clinical features of TB or sarcoidosis
- Absence of granulomatous disease history
- Medical record of extrathorasic malignancy
- Histopathological diagnosis of epithelioid histiocytes and/or multinucleated histiocytic giant cells, and/or nonnecrotizing granulomas
- Absence of alternative diagnosis

### 2.7. Inclusion criteria for pneumoconiosis

- Occupation history for pneumoconiosis
- Radiological findings consistent with pneumoconiosis
- Absence of alternative diagnosis

### 2.8. Inclusion criteria for nonspecific granulomas

Patients who do not meet the above criteria and have no history of malignancy or drug-related history were considered to have nonspecific granulomas.

## 3. Statistical analysis

The data were analyzed using SPSS for Windows 20.0 package program. First, descriptive statistics were presented. Ratio comparisons in tables (e.g., 2 × 2, 3 × 2) were performed using the chi-square test. Normality analysis of continuous data was performed using the Shapiro–Wilk test. For normally distributed data, the mean and standard deviation are presented; for data that did not show a normal distribution, the median, interquartile range, and minimum/maximum values are presented. Comparison of continuous data (not normally distributed) between more than two groups was performed using the Kruskal–Wallis test. A p-value of <0.05 was used for statistical significance.

## 4. Results

**Figure 1:**
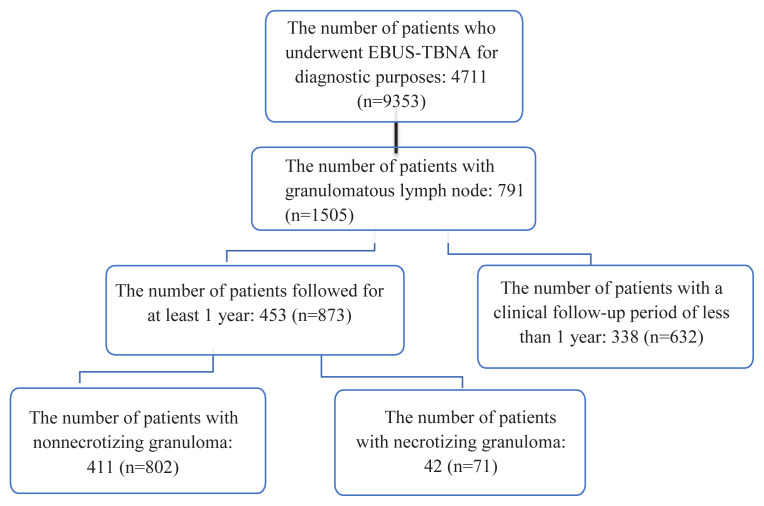
Patient count and lymph node sampling in EBUS-TBNA performed for diagnostic purposes. (n: number of lymph nodes)

Between January 2013 and December 2020, patients who underwent mediastinal lymph node sampling via EBUS-TBNA were retrospectively reviewed. As shown in Figure 1, a total of 9353 lymph node samples were obtained from 4771 patients, of which 1505 showed granulomatous lymphadenitis. Among these, 453 patients were followed for 1 year, and 873 lymph node samples collected. The majority of the patients (69.1%) were female, and the mean age was 48 years (range: 18–82 years). The most frequently sampled lymph nodes were subcarinal (41.2%) and right lower paratracheal (20.7%) lymph nodes.

As shown in Table 1, granulomatous lymph nodes were categorized based on their ultrasonographic features. Homogeneous internal structures were observed in nonnecrotizing granulomatous lymphadenitis, while heterogeneous internal structures were seen in necrotizing granulomatous lymphadenitis (p < 0.001).

**Figure 2:**
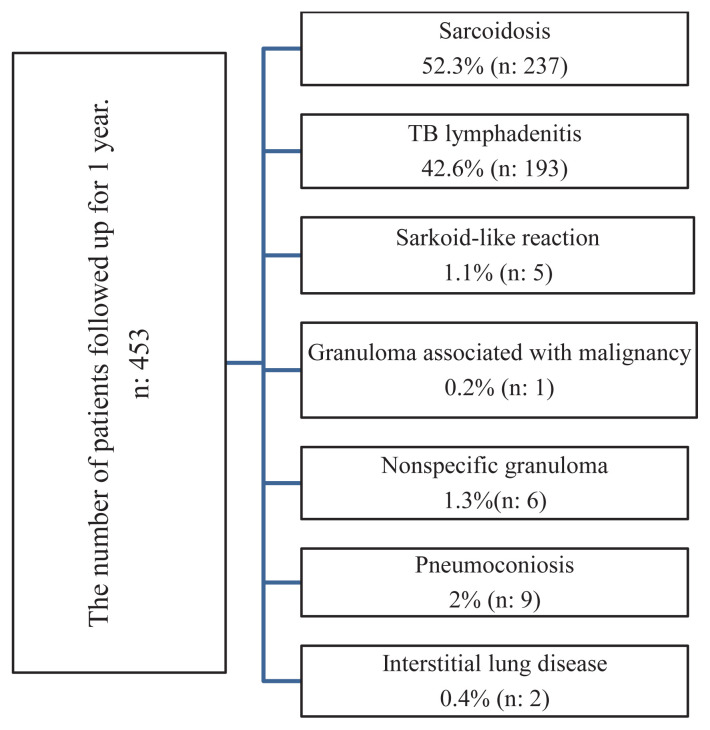
Etiological distribution after 1 year. (n: number of patients; TB: tuberculosis)

The final diagnoses after 1 year of monitoring were as follows: sarcoidosis in 52.3% (n = 237), tuberculosis (TB) lymphadenitis in 42.6% (n = 193), sarcoid-like reaction (SLR) in 1.1% (n = 5), Hodgkin lymphoma in 0.2% (n = 1), pneumoconiosis in 2% (n = 9), nonspecific granuloma in 1.3% (n = 6), and interstitial lung disease in 0.4% (n = 2) (Figure 2).

**Figure 3:**
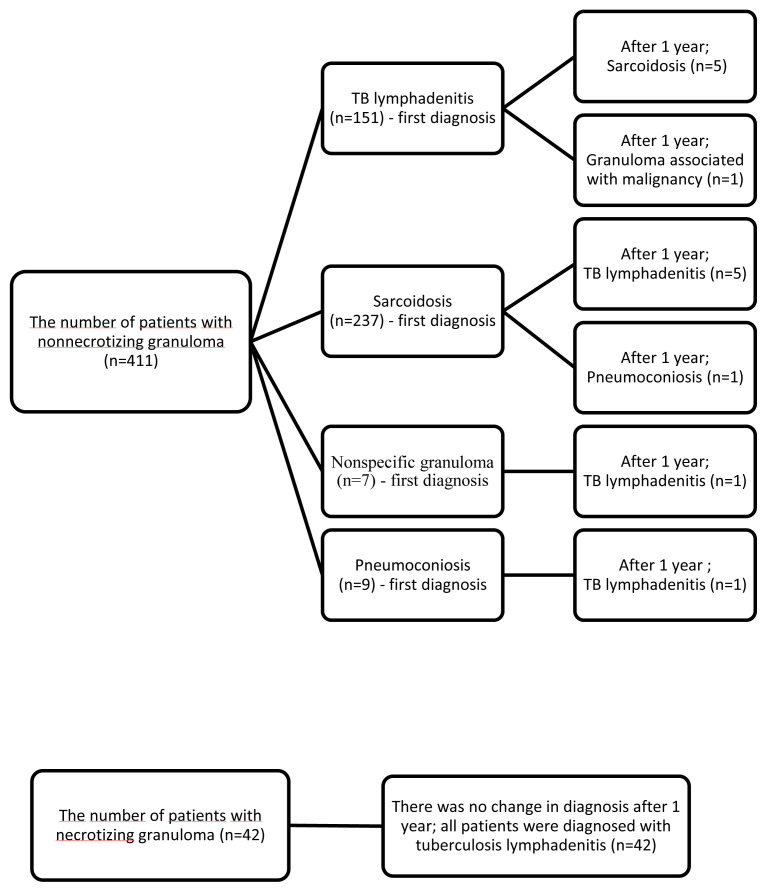
Patients with diagnostic changes after 1-year of follow-up. (n: number of patients; TB: tuberculosis)

As shown in Figure 3, the diagnosis changed in some patients after 1 year of follow-up. The histopathological findings of these patients were consistent with nonnecrotizing granulomatous lymphadenitis. AFB staining and cultures were negative, and there were no parenchymal findings on imaging. These patients were clinically monitored, and their treatment responses (such as methylprednisolone response for sarcoidosis and anti-TB response for TB) were evaluated. Biopsy was not repeated in patients who did not show improvement with treatment. Changes in diagnosis and treatment were discussed in multidisciplinary team meetings with radiologists, pathologists, and pulmonologists. In the only patient diagnosed with Hodgkin lymphoma, the diagnosis was confirmed by excisional biopsy of a cervical lymph node.

Among the 8 patients with a history of extrathoracic malignancy and nonnecrotizing granulomatous lymphadenitis, 5 were diagnosed with SLR and 3 with TB lymphadenitis. The extrathoracic malignancies causing sarcoid-like reactions were as follows: breast cancer (n = 2), colon cancer (n = 1), ovarian cancer (n = 1), and malignant melanoma (n = 1). No relapses were observed in SLR patients during the 1-year follow-up. These patients were not treated and were monitored clinically and radiologically.

As shown in Table 2, 94.3% of the patients’ EBUS-TBNA biopsy samples were tested for TB using AFB staining, PCR, and culture. Approximately 5.3% of these test results were positive.

## 5. Discussion

The aim of this study was to determine the etiological diagnoses and observe alterations in diagnosis following a minimum of 1 year of clinical follow-up for patients diagnosed with granulomatous lymphadenitis via endobronchial ultrasound-guided transbronchial needle aspiration (EBUS-TBNA) in a region endemic for tuberculosis.

In our study, after 1 year of clinical monitoring of 453 patients with granulomatous lymphadenitis sampled via EBUS-TBNA, the final diagnoses were determined as 52.3% (n = 237) sarcoidosis and 42.6% (n = 193) tuberculosis lymphadenitis. In a study by Erbay et al., among 110 patients with mediastinal/hilar granulomatous lymphadenitis, sarcoidosis was identified in 71.8%, TB in 3.6%, and idiopathic granulomatous lymphadenitis in 10.9% [1]. Another study reported that among 135 patients of granulomatous lymphadenitis, 113 (77.9%) were diagnosed with sarcoidosis and 22 (67.6%) with TB [6]. The higher prevalence of TB lymphadenitis compared to other studies is attributed to the fact that EBUS-TBNA sampling was performed not only on suspected sarcoidosis cases but on all mediastinal lymphadenopathy cases. In almost all patients diagnosed with granulomatous lymphadenitis, TB tests were conducted. Additionally, our center is one of the main referral hospitals for TB in Türkiye, which has contributed to the higher number of cases and explains the increased diagnosis of TB lymphadenitis compared to other studies.

SLR are nonnecrotizing granulomas observed in regional or draining lymph nodes of extrathoracic malignancies. They are histopathologically similar to sarcoidosis but can be clinically differentiated [7]. Several studies have shown that SLR has a high negative predictive value for metastasis [7–10]. In our study, the types of extrathoracic malignancies showing SLR were breast cancer, ovarian cancer, and malignant melanoma. However, although SLR can precede malignancy in rare cases, in our study, there were no size changes in lymph nodes after 1 year of monitoring [11]. In a study by Arslan et al., lymphoma was diagnosed in one patient and lung cancer in another after 3 years following the initial diagnosis of SLR [11]. In the study by Butt et al., recurrence was observed in 2 out of 26 patients with extrathoracic malignancies [9]. Grosu et al. found that in 5.3% of patients with extrathoracic malignancies and SLR, lymph nodes enlarged during follow-up [12].

In our study, the final diagnosis changed in 14 patients with granulomatous lymphadenitis after one year. All of these patients’ *Mycobacterium tuberculosis* smear, real-time PCR, and culture results were negative and they had nongranulomatous lymph nodes. The clinical characteristics of these patients were similar, and there were no parenchymal findings on imaging. The most common diagnostic change occurred among sarcoidosis and tuberculosis lymphadenitis. The decision of changing the diagnosis was made in multidisciplinary team meetings. In cases of single intrathoracic lymphadenitis, it can be challenging to differentiate TB from sarcoidosis, as there are significant similarities between the clinical, radiological, and cytopathological findings of these diagnoses [13]. Especially in TB endemic regions, it remains unclear whether the cytomorphological characteristics of granulomas can be used to differentiate TB and sarcoidosis. Gupta et al. conducted a study where a cytopathologist, without any clinical data, reevaluated EBUS-TBNA samples. In this study, 40 out of 135 granulomas (29.4%) detected in 75.4% of patients were misdiagnosed initially [6].

EBUS-TBNA is recommended for the diagnosis of granulomatous mediastinal lymphadenopathy due to its safety and minimally invasive nature [3].

In a study examining the diagnostic benefit of EBUS-TBNA for intrathoracic tuberculosis lymphadenitis in patients with mediastinal or hilar lymphadenopathy of unknown etiology, the sensitivity of EBUS-TBNA was calculated to be 59.3% [13]. Another study found sensitivity and accuracy above 60% [3].

In a metaanalysis of seven studies evaluating the diagnostic efficacy of EBUS-TBNA for tuberculosis lymphadenitis, Ye et al. found that the sensitivity was 0.80 (95% CI, 0.74–0.85) and the specificity was 1.0 (95% CI, 0.99–1.00) [14]. These studies highlight the reliability of EBUS-TBNA in terms of specificity, while also demonstrating limitations in its sensitivity. To minimize these limitations, especially in tuberculosis-endemic regions, it is recommended to perform mycobacterial sampling on EBUS-TBNA specimens and to consider the increased sensitivity and specificity of TB-PCR testing [15,16]. We emphasize the importance of using TB-PCR testing. Additionally, due to the limitations of cytomorphological features in providing a definitive diagnosis, long-term clinical monitoring and evaluation of the treatment response play a significant role in the diagnostic process.

The limitations of the study include the 1-year duration of clinical follow-up, which may be insufficient to detect long-term diagnostic changes. Furthermore, due to the retrospective design of the study, PET-CT results and thoracic CT findings were not available for all patients.

## 6. Conclusion

In regions where tuberculosis is endemic, the possibility that the final diagnosis may change should be considered in patients diagnosed with granulomatous lymphadenitis, and clinical follow-up should be conducted for at least 1 year. AFB staining and TB-PCR testing should be performed on EBUS-TBNA material.

## Figures and Tables

**Table 1:** Ultrasonographic features of granulomatous lymph nodes.

Granulomatous lymphadenitis		Nonnecrotizing granulomatous lymphadenitis		Necrotizing granulomatous lymphadenitis		p-value
		Count	Column N %	Count	Column N %	
Echogenicity	Anechoic	1	0.1%	0	0.0%	-
	Hypoechoic	800	99.8%	70	100.0%	
	Isoechoic	1	0.1%	0	0.0%	
İnternal structure	Homogeneous	486	60.6%	14	20.0%	<0.001
	Heterogeneous	316	39.4%	56	80.0%	
Shape	Oval	463	57.7%	27	38.6%	-
	Round	332	41.3%	42	60.0%	
	Triangle	9	0.9%	1	1.4%	
Borders	Clear	772	96.1%	66	94.3%	-
	Unclear	31	3.9%	4	5.7%	
Calcification	No	771	96.0%	67	95.7%	-
	Central	13	1.6%	3	4.3%	
	Eccentric	19	2.4%	0	0.0%	
Pearson chi-square test						

**Table 2:** EBUS-TBNA biopsy material; *Mycobacterium Tuberculosis* smear, real-time PCR, and culture results.

Smear, real-time PCR, and culture results	Count	N%
All negative (smear, real-time PCR, and culture)	403	89.0%
Smear negative, real-time PCR positive, and culture negative	4	0.9%
Smear negative, real-time PCR negative, and culture positive	20	4.4%
Number of patients whose samples were not sent	26	5.7%
PCR: Polymerase chain reaction		

## Footnotes

1 World Health Organization (2025). Tuberculosis surveillance and monitoring in Europe [online]. https://www.ecdc.europa.eu/sites/default/files/documents/TB-2025-Surveillance-report.pdf [Accessed 15 April 2025].
